# Antioxidant Activity and Glucose Diffusion Relationship of Traditional Medicinal Antihyperglycemic Plant Extracts

**Published:** 2013

**Authors:** Fariba Asgharpour, Mahdi Pouramir, Asieh Khalilpour, Sobgol Asgharpour Alamdar, Mehrasa Rezaei

**Affiliations:** 1 *Paramedical Faculty, Babol University of Medical Scienes, Babol, Iran.*; 2 *Cellular and Molecular Biology Research Center (CMBRC), Babol University of Medical Scienes, Babol, Iran.*; 3 *Paramedical Faculty, Babol University of Medical Scienes, Babol, Iran.*; 4 *Bureau of Education, Rasht-Region 2, Rasht, Iran.*; 5 *Faculty ofVeterinary MedicineUniversity of Urumieh, Urumieh,Iran.*

**Keywords:** Antihyperglycemic plants, glucose diffusion, antioxidant activity, polyphenols, flavonoids

## Abstract

Plants with hypoglycemic properties are important in the treatment of diabetes. One of the mechanisms in reducing blood glucose is preventing the digestive absorption of glucose. The aim of this study was to evaluate the antioxidant properties of some traditional medicinal plants collected from different regions of Iran and their effects on glucose diffusion decrease. The amounts of phenolic compounds, total flavonoids, total polysaccharides, antioxidant activity and lipid peroxidation were determined respectively by folin ciocalteu, querceting, sulfuric acid, FRAP and thiobarbituric acid - reactive substanses (TBARS) in eleven confirmed traditional antihyperglycemic medicinal plants prepared at 50g/l concentrations using the boiling method. Phenolic compounds of *Eucalyptus globules* (100.8± 0.01 mg /g), total flavonoids content of *Juglans regia* (16.9± 0.01 mg /g) and total polysaccharide amount of *Allium satirum *(0.28± 0.05) were the highest. Significant relationship was observed between the polyphenols and flavonoids (p <0.05). The grape seed extract showed the highest antioxidant activity (133± 0.02 mg/g) together with decreased glucose diffusion as well as increased polyphenols (p <0.05), but the increase in antioxidant activity was not related to glucose diffusion. Antihyperglycemic plant extracts containing higher polyphenols showed more efficiently *in vitro* glucose diffusion decrease, but no significant relationship was observed between antioxidant activity increase and glucose diffusion.

Reactive Oxygen species (ROS) including radicals of superoxide, hydroxyl and peroxyl are produced continuously in our body. These radicals have an important role in oxidative stress occurrence causing progress toward different diseases (1).

Flavonoids and other phenol compounds are secondary metabolites present in plants. Phenol compounds have physiologic properties including anti allergic, anti-microbial, anti-coagulant, anti-inflammation and conservation effect. They also have a beneficial role for coronary disease management as well as cancer and neuro-degenerative diseases prevention (2-3). The beneficial effects of phenol compounds are related to their antioxidant activities. Most researches in last decades evaluated the chemistry of phenol compounds, their antioxidant activities, the existence of these compounds in food and non-food sources, their bioavailability, metabolism and also their potential as a food antioxidant (4). The antioxidant effect of these components is due to their reducing properties, their ability to act as hydrogen donor and chelate metals. Some investigations showed that foods containing antioxidants have useful role in the maintenance and promotion of heath. For example, there is an inverse relationship between the occurrence of coronary artery disease and cancer and the amount of polyphenol content in foods. Many studies were performed in order to find antioxidant molecules in natural resources (5-6). Wash et al. (2005) reported that the sources of phenols and flavonoids supply in different parts of the world depend on the diet of those regions. In countries like Japan and China, green tea usage could provide necessary phenolic compounds of the body, but these compounds are provided by consuming apple and onion in western countries, vegetables and fermented food materials in eastern countries (7). In Iran high consumption of vegetables and fruits, provide vitamins and minerals and can be a good source of natural antioxidants (8).

The ferric reducing of antioxidant power (FRAP) assay was used previously for the determination of total antioxidant power of eleven traditional medicinal plants in our laboratory. FRAP assay is an acceptable method for determining the total antioxidant power of biological samples (9-10). In fact using this method, it is possible to measure the reducing power of antioxidants such as vitamin C, vitamin E, flavonoids etc. This method is also very accurate and fast (11). Today other methods are used for measuring the power of antioxidants which are based on the neutralization of free radicals by available antioxidants in biological samples (10). But due to the use of chemical reactions and the mechanism that represents an estimate of the antioxidant capacity of the samples, all these methods are performed *in vitro* (12).

In our previous study (13), hypoglycemic plant extracts (50 g/l) showed reduced glucose diffusion from dialysis bag and their AUC (area under curves) in comparison to control, after 24 h. The aim of the present study was to evaluate the antioxidant activity of these traditional medicinal plants and their relationship to glucose diffusion decrease.

## Materials and Methods

**Plant materials**

Eleven traditional antihyperglycemic plants (*Securigera securidaca, Citrullus colocynthis, Coriandrum satirum, Allium satirum, Salvia officinal, Eucalyptus globules, Urtica dioica, Juglans regia, Vitis vinifera, Viscum album, Pyrus biosseriana*) were collected and their authenticity was confirmed by Mazandaran Agricultural and Natural Resources Research Center, Mazandaran, Iran.

**Preparation of extracts**

Aqueous extracts were prepared from the traditional plants using decoction method. One gram of powdered material was added to 40 ml of distilled water and allowed to boil for 15 min. Each suspension was filtered (whatman no.1) and the volume was readjusted to 40 ml with distilled water. The provided extracts were dried at 45°C for 24- 48 h (9). The concentration of 50g/l of aqueous extracts was used in all the experiments.

**Glucose diffusion Test**

8 cm × 6.4 mm dialysis bags (spectra/ por, MWCO: 500- 1000) were used. 2 ml of each extract in 15M NaCl and 22 M D - glucose were added to dialysis bags which were then closed and placed into a 50 mL tube containing 45 mL of 15M NaCl. Glucose concentrations were measured at 2 hours time intervals and compared to the control (13).

**Total phenolics content**

Folin ciocalteu reagent use was adapted from McDonald to determine total phenolics content (14). The diluted extract (0.5ml of 1:10, v/v) and phenolic standard were mixed with Folin Ciocalteu reagent (5ml, 1:10 diluted) and aqueous Na2CO3 (4ml, 1M). Solutions were heated for 15 min in a water bath at 45°C and the total phenols were determined spectrophotometrically at 765 nm. The standard curve was prepared using 0, 50, 100, 150, 200, 250 and 500 mg/L solutions of gallic acid in methanol: water (50:50, v/v). Total phenol values are expressed as gallic acid equivalents (mg/g dry weight).

**Total flavonoids content**

Total flavonoid content was determined using aluminum chloride and photometry (15).The plant extract (0.5 mL of 1:10 g/mL) in methanol was mixed with 1.5 ml methanol and 0.1 ml of aluminum chloride (1% ), 0.1 ml potassium acetate (1 M) and 2.8 ml distilled water. After 30 min incubation at room temperature, sample absorbance was read at 415 nm. Querceting solutions calibration curve was prepared in the range of 0-50 mg/ ml in methanol. Results are reported as quercetin equivalent, mg / g dry weight.

**Total polysaccharides content**

Total polysaccharides were measured by sulfuric acid method (16). In this experiment, the glucose )10- 100 µg/l) was used as standard; 1 mL of the extract was added to 1 mL of phenol solution (5% w/v) and 2 ml concentrated sulfuric acid (98-95%). The absorption of samples was determined at 470 nm by spectrophotometer (UV-Visible). Results are reported in mg/g dry weight.

**Total antioxidant activity assay**

Total antioxidant activity was estimated by a FRAP assay (17). The FRAP reagent contained 2.5 ml of a 10 mmol/L TPTZ (2, 4, 6-tripyridyl-s-triazine; Sigma) solution in 40 mmol/L HCl plus 2.5 ml of 20 mmol/L FeCl3 and 25 ml of 0.3 mol/L acetate buffer (pH 3.6). The reagent was freshly prepared and warmed at 37 °C. The working FRAP reagent (1.5 ml) was mixed with 50 μl sample or standard in a test tube. After 10 min at 37 °C, the absorbance was determined at 593 nm. FeSO4 at a concentration of 1 mmol/L was used as the standard solution. The final result was expressed as the concentration of antioxidant with a ferric reducing ability equivalent to that of 1 mmol/l FeSO4.

**TBARS Test**

In this method, 4ml of TBA /TCA and 50 µl of BHT were added to all tubes and placed in boiling bath for 15 min. Tubes were centrifuged for 15 min at 3000 rpm and the absorption of surfactant was read at 532 nm by spectrophotometer (18).

**Statistical Analysis**

All measurements were repeated 3 times and data were reported as Mean± SD. Pearson correlation analysis was used and P<0.05 was considered as significant. SPSS software Version 17 was used for statistical analyses.

## Results

**Fig. 1:**
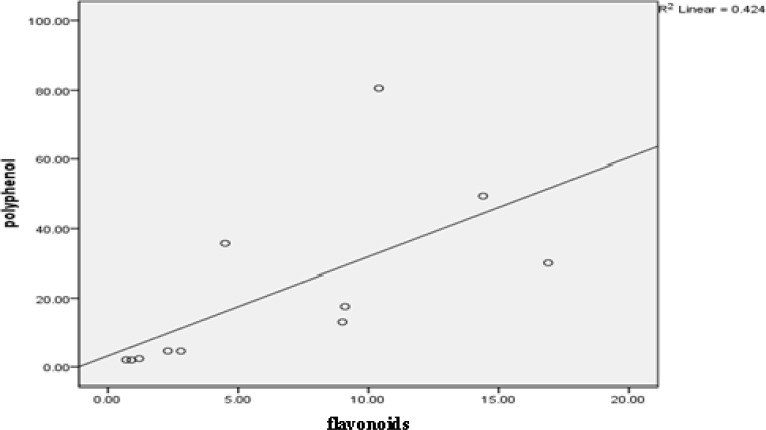
Relationship between flavonoids and polyphenols concentration (P< 0.05).

The amount of phenolic complex was between (2-80.2mg/g). Among the analyzed extracts, *Eucalyptus* *globules*, *Vitis* *vinifera* and *Securigera securidaca* had the highest amounts of phenolic contents and *Viscum album*, *Citrullus colocynthis *and *Allium satirum* had the lowest values of Polyphenols. Aqueous extracts of *Juglans* *regia*, *Vitis* *vinifera* and *Eucalyptus* *globules* had the maximum concentration of flavonoids whereas *Citrullus colocynthis,* *Allium satirum* and *Salvia officinal* had the minimum concentration of flavonoids. Aqueous extracts of Allium satirum,* Vitis vinifera* and *Securigera securidaca* had the highest concentration of polysaccharides (Table 1). Also, a significant relationship (p<0.05) was found between polyphenols and flavonoids (Fig. 1).

**Fig. 2:**
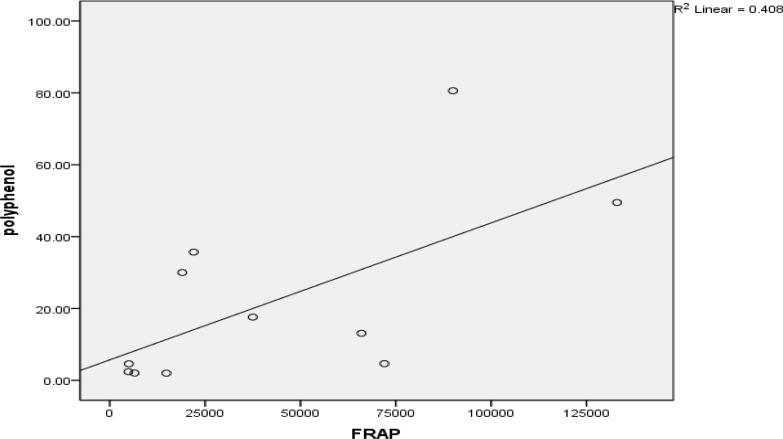
Relationship between flavonoids and polyphenols concentration (P< 0.05).

The highest antioxidant activity was present in* Vitis* *vinifera*, *Eucalyptus* *globules* and *Pyrus biosseriana* and the lowest antioxidant activity was observed in *Urtica* *dioica*, *Citrullus colocynthis* and *Viscum album*. Among these medicinal plants, *Eucalyptus* *globules* and *Vitis vinifera* had a high antioxidant power, polyphenols and flavonoids. But some medicinal plants such as *Juglans* *regia* and *Securigera securidaca* that had high amounts of polyphenols and flavonoids did not show high antioxidant activities. A significant relationship (p<0.05) was found between polyphenols and FRAP assay results (Fig. 2).

## Discussion

The results of this study showed that some medicinal plants have very high antioxidant activity. Similar studies in different countries evaluated the antioxidant power of medicinal plant extracts by FRAP method including the 27 types of fruits in Singapore (19), 45 medicinal plants in Cuba (20), 34 types of vegetables and 30 types of fruits in Italy (21) and also 28 medicinal plants in Iran (22). In the present study, *Vitis* *vinifera* showed more antioxidant activities than other plant extracts examined (133± 0.02 mg/g) and *Viscum album* had the lowest antioxidant activities (4.9± 0.06 mg/g).

**Table 1:** Antioxidant activity (FRAP), Thiobarbituric acid- reactive substances (TBARS), polyphenols, flavonoids, Polysaccharides concentrations and Area under curves (AUC) in eleven traditional antihyper-glycemic plants (Mean ± SD)

**Plant extract **	**Test**					
	Polysaccharide (mg/g)	polyphenol (mg/g)	Flavonoid (mg/g)	FRAP (Mm/L)	TBARS (μM/L)	AUC*
*Securigera securidaca*	0.22± 0.09	35.70± 0.01	4.50± 0.00	22.00± 0.09	2.37± 0.02	33.50± 0.50
*Citrullus colocynthis*	0.04± 0.02	2.04± 0.03	0.70± 0.06	6.50± 0.01	1.87± 0.02	53.70± 3.00
*Coriandrum satirum*	0.09± 0.08	13.10± 0.01	9.00± 0.02	66.00± 0.03	2.29± 0.01	38.40± 0.30
*Allium satirum*	0.28± 0.05	2.00± 0.00	0.90± 0.04	14.90± 0.01	1.55± 0.01	75.50± 7.00
*Salvia officinal*	0.14± 0.02	17.60± 0.04	9.10± 0.04	37.50± 0.02	2.29± 0.002	66.20± 6.00
*Eucalyptus globules*	0.02± 0.01	80.60± 0.01	10.40± 0.04	90.00± 0.02	1.79± 0.01	18.60± 0.60
*Urtica dioica*	0.12± 0.04	4.60± 0. 08	2.80± 0.02	5.00± 0.04	3.44± 0.004	54.30± 3.00
*Juglans regia*	0.16± 0.04	30.00 ± 0.00	16.90± 0.02	19.00± 0.05	2.25± 0.07	50.50± 0.60
*Vitis * *vinifera*	0.22± 0.01	49.50± 0. 02	14.40± 0.02	133.00± 0.02	3.65± 0.00	44.60± 0.30
*Viscum album*	0.04± 0.02	2.40± 0.01	9.10± 0.04	4.90± 0.06	1.27± 0.01	55.00± 0.30
*Pyrus biosseriana*	0.11± 0.02	4.60± 0.01	2.30± 0.01	72.00± 0.10	2.71± 0.04	43.70± 1.00

* AUC was measured in a previous study.

Aqueous extracts of *Eucalyptus globules* and *Allium satirum* had the highest and lowest polyphenols contents, respectively. *Juglans regia* leaf extract had the most flavonoids content, but did not show a higher antioxidation activity than other plant extracts. Also the aqueous extract of *Allium satirum* had the most poly-saccharides content but the amount of polyphenols and flavonoids as well as antioxidant acitivity were not high. 

**Fig. 3:**
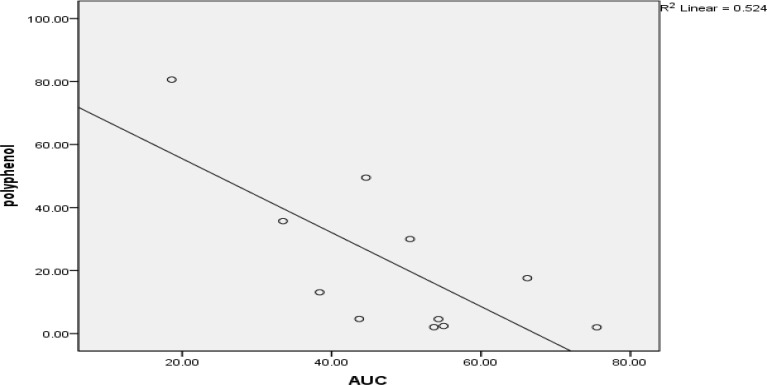
Relationship between AUC (Area under curve) and polyphenols concentration (P< 0.05).

According to other studies, the antioxidant activities of most extracts were related to available polyphenols (8). Aqueous extract of *Vitis* *vinifera* and *Eucalyptus globules* that showed a high amount of polyphenols and flavonoids, had also high antioxidant activities which can be attributed to the presence of those compounds. These compounds consist of monomer flavonoids including catechin, epicatchin, dimer flavonoid, trimer flavonoid, polymer prosiyanidin and gallic acid and ellagic acid (23). Researches showed that proantisyanin in grape seed has antioxidation properties (23). In a study reported by Oberley et al. (2005), it was demonstrated that this compound has also an effect on cardiovascular disease (24). In another study, red grape seeds extracts caused a significant reduction in lipid oxidative damage in the brain, liver and gastrointestinal mucosa in diabetic animals (25). Grape seed in high doses has been efficient in decreasing hyperglycemia induced by alloxan injection, probably as a result of its antioxidant activity (23). The antioxidation properties of grape seeds can be related to other compounds that prevent harmful effects of alloxan and therefore decrease glucose levels (26). In a previous study, aqueous extract of grape seeds at 50g/1 concentration prevented 57% of glucose diffusion from dialysis bag (13).

In this study, it was also observed that aqueous extract of *Eucalyptus globules* has high polyphenols and antioxidation activity. In a study performed by Amakura et al. (2007), the main reason for higher antioxidant activities in *Eucalyptus globules* leaves was the presence of gallic acid and ellagic acid (27). In Japan, this plant's leaves extract as a natural source of antioxidants is among the foods additives list (27). (28). Photochemical analysis of *Eucalyptus* showed the presence of components including monoterpen (29), cyanogenic glycosides (30), tripens and cladocalol (31). The results of our previous study showed that the aqueous extract of *Eucalyptus* *globules* had a lower viscosity than other examined extracts and the highest glucose diffusion property (13). The aqueous extracts of some medicinal plants such as Juglans regia that have high amount of flavonoids (16.9± 0.02 mg/g) did not show higher antioxidation activities (19± 0.05 mM). *Juglans* *regia* leaves contain significant amounts of antioxidants such as phenolic compounds. Its leaves have 3 percent of materials including Inojit, alagic acid, gallic acid and some amount of paraffin, tanon, fatty material and minerals like K, P, Mg, Br and carotene. Also these leaves are used for diabetic cure in Iranian traditional medicine (32). In Zarban study on *Juglans* *regia*, the aqueous extract of this leaf together with aqueous extracts of Viscum album and aqueous and alcoholic extracts of *Salvia officinal* had the highest antioxidant activity (22). To compare the antioxidant activities of the aqueous extracts of *Allium satirum, Salvia officinal, Juglans regia, Coriandrum satirum* and *Viscum album*, the highest antioxidant activities in Zarban study were observed in *Salvia officinal* and *Viscum Album* while aqueous extracts of *Coriandrum satirum* and *Allium satirum* showed the lowest antioxidant activities (22). In this study, among these plants, the aqueous extracts of *Coriandrum satirum* and *Viscum album* showed the highest and lowest antioxidant activities, respectively. However, it should be considered that seasonal and climate changes, different geogra-phical areas, conditions of plant growth and different species of plants parameters may influence qualitatively and quantitatively the results. Some components may change to other compounds, leading either to an increase or a decrease of their quality and quantity. For example, *Viscum Album* is a semi-parasitic plant species that usually grows on trees like *Apples, Pyrus, Ulmus minor, Crataegus aronia* etc… The growth of this plant on different trees and different feeding and growth conditions can affect the active components of this plant.

In this study, the aqueous extract of *Allium satirum* had more polysaccharides than other examined medicinal extracts but the amounts of polyphenols and flavonoids and antioxidant acitivity were not high. In Zarban’s study, the aqueous and alcoholic extracts of *Allium satirum* had the lowest antioxidant activities (22). The aqueous extract had a higher antioxidant activity than the alcoholic extract. This may be due to a better solubility of different antioxidants in aqueous solvents (33). For a better evaluation of the effects of these plant extracts, it is necessary to examine the side effects of long term use and also evaluate the antioxidant properties of medicinal plants in laboratory animals or humans.
